# Increased Risk for Multidrug-Resistant Tuberculosis in Migratory Workers, Armenia

**DOI:** 10.3201/eid2103.140474

**Published:** 2015-03

**Authors:** Nune Truzyan, Byron Crape, Ruzanna Grigoryan, Hripsime Martirosyan, Varduhi Petrosyan

**Affiliations:** American University of Armenia School of Public Health, Yerevan, Armenia

**Keywords:** tuberculosis and other mycobacteria, multidrug-resistant tuberculosis, MDR TB, risk reduction behavior, medication adherence, migrant workers, Armenia

## Abstract

To understand use of tuberculosis (TB) services for migrant workers, we conducted a cross-sectional census of 95 migrant workers with TB from Armenia by using medical record reviews and face-to-face interviews. Prolonged time between diagnosis and treatment, treatment interruption, and treatment defaults caused by migrant work might increase the risk for multidrug-resistant TB.

Economic transition after the breakup of the former Soviet Union pushed many Armenians to look for new job opportunities in other countries (*1**–**3*). A total of 15.0% of Armenian families include migrant workers who regularly seek seasonal work in host countries and return on completion of that work (*1**,**2*). Armenia is a country in the southern Caucasus and has a population of 3 million persons (*4*) who face a reemerging threat from tuberculosis (TB). According to the National TB Control Program, the incidence of TB/100,000 persons increased from 16.6/10,000 persons in 1990 to 62.4/100,00 persons in 2005 (*5*). This threat is exacerbated by increased rates of drug-resistant TB (*6*).

Among newly diagnosed TB cases in Armenia, ≈9.4% are multidrug-resistant TB (MDR TB), and 43.0% of previously treated TB cases become MDR TB cases (*5**–**7*). Armenia reported 7 cases of extensively drug-resistant TB in 2011 (*5*). The purpose of this study was to evaluate how characteristics of migrant workers influence use of TB services in the host country of work and in the country of origin and TB treatment outcomes. 

## The Study

Study protocols were approved by the American University of Armenia Institutional Review Board. Given the lack of data on TB among migrant workers, we contacted TB physicians working in all 72 outpatient TB centers in Armenia. The attending TB physicians were familiar with the migrant worker status of their patients, and a list of their patients formed a census study frame for all migrant workers with TB. All members of the study population had a diagnosis of TB. A study participant had to be a migrant worker outside Armenia for ≥3 months during 2008–2011, be ≥18 years of age, have Armenian citizenship, and speak Armenian. We collected data from eligible study participants in the census study time frame through face-to-face interviews and abstraction of data from medical records. After considering national data on TB prevalence in Armenia and the number of migrant workers with a TB diagnosis provided by TB physicians and confirmed during the study, we estimated that ≈7.0% of all TB patients treated during 2008–2011 in Armenia were migrant workers.

We attempted to contact all 126 eligible migrant workers in Armenia at the time of the survey (January–March 2012) and recruited 95 study participants (75.0% response rate). Approximately 91.0% of study participants had either a high school or professional technical education, 85.0% were from low-income households, and ≈33.0% were registered in a social support program for persons living in poverty. Median ages of surveyed persons and the population with TB in Armenia in 2012 were similar (46 and 45 years, respectively). The 95 study participants reported 166 visits for seasonal work to other countries during 2008–2011; the Russian Federation was the most common destination (147 visits) (Figure). The type of work in which study participants were involved outside Armenia was construction (65.0%), driving/transportation (11.0%), commerce (8.0%), production (7.0%), services (5.0%), food industry (4.0%), and farming (1.0%). For ≈63.0% of all host country work visits, work was in confined spaces, of which 23.0% had >6 persons working in the same enclosed space. For 7.0% of all visits, respondents reportedly resided in overcrowded conditions.

**Figure:**
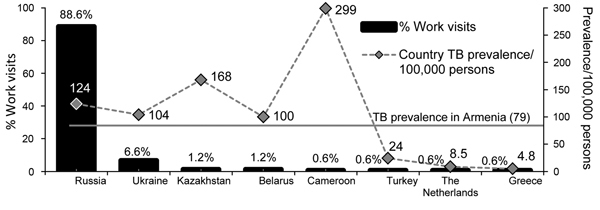
Percentage of work visits by migrant workers to host countries-of-work and tuberculosis (TB) prevalence/100,000 persons in host countries compared with that in Armenia, 2008–2011 (*7*).

Medical record reviews of 95 study participants indicated that 28.3% (26/92) of respondents had MDR TB and 5.3% (5/94) had HIV/TB co-infection compared with 5.6% and 3.1%, respectively, for all TB patients in Armenia reported by the National TB Control Program in 2011 (Table 1). All study participants were treated for TB at least once, 73.6% (70/95) were reportedly treated only once, 13.7% (13/95) were treated twice, 12.6% (12/95) were treated ≥3 times, and 44.2% (42/95) of migrant workers were reportedly first given a diagnosis of TB in the host country of work.

**Table 1:** Characteristics of 126 migrant workers and all TB patients in Armenia, 2008–2011*

Characteristic	No. positive/ no. tested (%)		Year, no. (%)			
	Survey participants, n = 95	Refused to participate, n = 31	2008, n = 2,125	2009, n = 2,006	2010, n = 1,780	2011, n = 1,584
HIV/TB co-infection	5/94 (5.3)	1/30 (3.2)	12 (0.6)†	17 (0.9)†	17 (1.0)†	49 (3.1)
MDR TB	26/92 (28.3)	5/31 (16.1)	77 (3.6)†	134 (6.7)†	154 (8.7)†	88 (5.6)†
Extrapulmonary TB	1/94 (1.1)	4/31 (12.9)†	380 (17.9)†	374 (18.6)†	429 (24.1)†	361 (22.8)†
SS+ at time of diagnosis	47/93 (50.5)	15/31 (48.4)	679 (32.0)†	561 (28.0)†	434 (24.4)†	420 (26.5)†

*MDR TB, multidrug-resistant tuberculosis; SS+, sputum smear positive. †Significant differences (p≤0.001) vs noninfected survey participants.

The period between first diagnosis and first treatment was reportedly ≈5 times longer (95% CI 1.9–34.8, p = 0.06) for those who were given a diagnosis in the host country of work than for those given a diagnosis in Armenia (Table 2). Approximately 92.5% (86/93) workers reported that they received inpatient hospital care during their first TB treatment, of whom 5 reportedly did not complete the full course of inpatient treatment. The mean duration for the first inpatient TB treatment for participants who received TB treatment in Armenia was 78 days (range 40–425 days). For those who received their first inpatient treatment in the host country of work, the mean duration was 164 days (range 20–912 days). Those workers who received treatment in the host country of work were 3.9 times more likely to have a failed or defaulted treatment outcome than those who received treatment in Armenia (95% CI 11.4–74.1, p = 0.001). However, only 13.7% (13/95) of respondents received treatment outside Armenia. Of those reporting, 9.3% (8/86) experienced an interruption of prescribed TB treatment for ≥1 day during the inpatient phase of their first treatment (mean duration of interruption 7 days, range 1–30 days).

**Table 2:** Time intervals between first symptoms, diagnosis, and tuberculosis treatment for migrant workers, Armenia, 2008–2011

Interval	Mean (SD) duration, mo		p value
	Armenia	Host country of work	
First symptoms–first diagnosis	3.1 (6.3)	1.7 (3.1)	0.23
First diagnosis–first treatment	0.6 (1.9)	3.1 (9.3)	0.06*
First symptoms–first treatment	3.8 (6.6)	3.8 (8.5)	0.54

*Marginally significant difference (0.1≤p≤0.05).

During the ambulatory phase of their first treatment, 20.4% (19/93) of respondents reported treatment interruptions (mean duration of longest interruption 7.8 days, range 1–60 days). Approximately 80.8% (55/68) of persons who completed their first treatment for TB reported that they completed the treatment (cured or completed treatment with no treatment failure), 11.7% (8/68) reported defaulting (lost-to-follow-up), and 7.4% (5/68) reported as being sputum-smear-positive at the end of treatment (treatment failed) (*8*). Bivariate analysis showed that patients who had incomplete ambulatory treatment had greater odds of having MDR TB than having drug-sensitive TB than did those who completed ambulatory treatment (odds ratio [OR] 4.0, 95% CI 1.6–10.1, p = 0.003).

Multivariate logistic regression showed that persons who did not receive standardized TB treatment with supervision during the ambulatory phase of treatment had 4 times greater odds (OR 4.4, 95% CI 1.3–14.9, p = 0.02) of having ≥2 TB treatments (versus only 1 TB treatment) than persons who received standardized treatment with supervision during the ambulatory phase. After adjusting for confounding, we found that persons who had MDR TB had 2 times greater odds (OR 2.3, 95% CI 1.1–5.0, p = 0.04) of having ≥2 TB treatments (versus only 1 TB treatment) than persons who had TB (Table 3).

**Table 3:** Associations between outcome variable of having 1 treatment versus multiple treatments for tuberculosis and potential risk factors for multiple treatments by bivariate and multivariate logistic regressions, for migrant workers, Armenia, 2008–2011*

Variable	Bivariate analysis		Multivariate analysis	
	Crude odds ratio (95% CI)	p value	Adjusted odds ratio (95% CI)	p value
DOT during ambulatory phase of first treatment				
Always or often	1.00	NA	1.00	NA
Sometimes or never	3.97 (1.2–13.3)	0.03†	4.36 (1.3–14.9)	0.02†
Having MDR TB				
No	1.00	NA	1.00	NA
Yes	2.11 (0.9–4.4)	0.05†	2.30 (1.1–5.0)	0.04†
Density of persons working in the same enclosed area in host country			NI	NI
1–5	1.00	NA		
≥6	0.15 (0.02–1.3)	0.08		
Crowding index for residence in host country			NI	NI
<2.0	1.00	NA		
≥2.0	1.68 (0.5–5.6)	0.39		
Crowding index for residence in Armenia			NI	NI
<2.0	1.00	NA		
≥2.0	1.28 (0.3–6.6)	0.77		
No. visits to the host country of work			NI	NI
1	1.00	NA		
>1	1.04 (0.4–2.6)	0.94		
Type of work performed in host country			NI	NI
Other	1.00	NA		
Construction	1.82 (0.7–4.9)	0.24		
Family member/relatives/friends having TB			NI	NI
No	1.00	NA		
Yes	0.84 (0.2–2.8)	0.77		
TB knowledge score			NI	NI
High (13–20)	1.00	NA		
Low (2–12)	1.30 (0.5–3.3)	0.58		
Education, y			NI	NI
>12	1.00	NA		
≤12	1.31 (0.5–3.4)	0.58		
Marital status			NI	NI
Married	1.00	NA		
Single/divorced/separated	0.99 (0.3–2.6)	0.84		
Wealth status			NI	NI
Not poor	1.00	NA		
Poor	2.38 (0.5–11.5)	0.28		

*DOT, directly observed therapy; NA, not applicable; MDR TB, multidrug-resistant tuberculosis; NI, not included. †Significant association (p≤0.05).

## Conclusions

We found that among migrant workers with a diagnosis of TB, migratory work was associated with higher rates of MDR TB and HIV co-infection, which suggested that migratory work may provide impetus for spread of HIV infection and TB. Treatment for TB that started in the host country of work was usually interrupted because migrant workers wanted to return to Armenia. Some of these workers did not resume treatment for TB in Armenia. Those migrant workers with TB who experienced treatment delays, dropped out of therapy, or had therapy interrupted were likely to increase the period of infectivity and spread TB to other persons.

There is no official referral system between the Armenian National TB Program and their counterparts in host countries of work. On the basis of our study findings, we recommend establishing closer collaboration between health systems of countries supplying migratory workers and host countries for work at governmental and nongovernmental levels to improve treatment completion rates and reduce adverse outcomes. This study could become a basis for further efforts in understanding the mechanisms that impede access of immigrants to treatment and planning target strategies for early detection of TB and treatment in this group of patients. It could also become an example for further large-scale projects worldwide, especially in the Commonwealth of Independent States region to explore the correlation between migrant labor and TB.
